# Tightly Regulated and Homogeneous Transgene Expression in Human Adipose-Derived Mesenchymal Stem Cells by Lentivirus with Tet-Off System

**DOI:** 10.1371/journal.pone.0066274

**Published:** 2013-06-12

**Authors:** Hiroyuki Moriyama, Mariko Moriyama, Kei Sawaragi, Hanayuki Okura, Akihiro Ichinose, Akifumi Matsuyama, Takao Hayakawa

**Affiliations:** 1 Pharmaceutical Research and Technology Institute, Kinki University, Higashi-Osaka, Osaka, Japan; 2 Platform for Realization of Regenerative Medicine, Foundation for Biomedical Research and Innovation, Chuo-ku, Kobe, Hyogo, Japan; 3 Department of Plastic Surgery, Kobe University Hospital, Chuo-ku, Kobe, Hyogo, Japan; Universidade de Sao Paulo, Brazil

## Abstract

Genetic modification of human adipose tissue–derived multilineage progenitor cells (hADMPCs) is highly valuable for their exploitation in therapeutic applications. Here, we have developed a novel single tet-off lentiviral vector platform. This vector combines (1) a modified tetracycline (tet)-response element composite promoter, (2) a multi-cistronic strategy to express an improved version of the tet-controlled transactivator and the blasticidin resistance gene under the control of a ubiquitous promoter, and (3) acceptor sites for easy recombination cloning of the gene of interest. In the present study, we used the cytomegalovirus (CMV) or the elongation factor 1 α (EF-1α) promoter as the ubiquitous promoter, and EGFP was introduced as the gene of interest. hADMPCs transduced with a lentiviral vector carrying either the CMV promoter or the EF-1α promoter were effectively selected by blasticidin without affecting their stem cell properties, and EGFP expression was strictly regulated by doxycycline (Dox) treatment in these cells. However, the single tet-off lentiviral vector carrying the EF-1α promoter provided more homogenous expression of EGFP in hADMPCs. Intriguingly, differentiated cells from these Dox-responsive cell lines constitutively expressed EGFP only in the absence of Dox. This single tet-off lentiviral vector thus provides an important tool for applied research on hADMPCs.

## Introduction

Human adipose tissue–derived mesenchymal stem cells (MSCs), also referred to as human adipose tissue–derived multilineage progenitor cells (hADMPCs), are multipotent stem cells that can differentiate into various types of cells, including hepatocytes [1], cardiomyoblasts [2], pancreatic cells [3], and neuronal cells [4]–[6]. They can be easily and safely obtained from lipoaspirates without posing serious ethical issues and can also be expanded ex vivo under appropriate culture conditions. Moreover, MSCs, including hADMPCs, have the ability to migrate to injured areas and secrete a wide variety of cytokines and growth factors necessary for tissue regeneration [7]–[11]. Because of their hypoimmunogenicity and immune modulatory effects, hADMPCs are good candidates for gene delivery vehicles for therapeutic purposes [12]. Thus, hADMPCs are an attractive material for cell therapy and tissue engineering, making the development of technologies for permanent and highly controlled genetic modification of hADMPCs quite valuable.

Lentiviral vectors are powerful tools for gene transfer in primary human cells, as they integrate into the host cell genome, resulting in stable long-term transgene expression. Lentiviral vectors are less prone to transcriptional silencing than oncoretroviral vectors [13], [14]; however, researchers have reported that transgene silencing occurs when a strong promoter, such as the cytomegalovirus (CMV) promoter, is used in certain cell types, especially embryonic stem cells [15]–[17]. Recently, it has been reported that the CMV promoter is also silenced in rat bone marrow–derived MSCs [18], [19], suggesting that consideration of promoter used in the lentiviral vector is one of the most critical issues.

In addition to the choice of promoters, the specific gene expression system can have a great impact on the properties and functions of the infected hADMPCs. In order to express therapeutic genes, master regulatory genes, or microRNAs, the development of a tightly regulated, inducible gene expression system is required. The tetracycline (tet)-regulated transgene expression (tet-off) system is the most advanced system being used in gene therapy trials [20]. Two expression cassettes need to be delivered for use of the tet-off system: the regulatory unit for the constitutive expression of the transactivator (tTA), and the tet-controlled responsive unit for the expression of the gene of interest. Traditionally, these 2 cassettes should be transduced separately to establish tet-inducible cell lines. This time-consuming process significantly limits the number of cell lines that can be generated for target gene expression. Recently, several researchers attempted to develop single-vector–based tet-inducible lentiviral systems [21]–[24]. However, the large plasmid size and lack of antibiotic selectable markers in these systems made the generation of plasmid constructs, high titer lentiviral particles, and stably expressing transgenic cell lines difficult.

To overcome the limitations of the current single vector-based tet-inducible lentiviral systems, we generated a robust system that incorporates all the necessary components for tet-off gene expression, restriction enzyme treatment/ligation independent cloning system, and antibiotic selectable markers in a single lentiviral vector. This vector consists of a modified tet-response element composite promoter (TRE-Tight) followed by a Gateway cassette containing *att*R recombination sites flanking a *ccd*B gene and a chloramphenicol resistant gene, which allows for easy and rapid shuttling of the gene of interest into the vector. This vector also carries an improved version of the tet-controlled transactivator (tTA-advanced) and the blasticidin resistance gene, linked by the self-cleaving viral T2A peptide, under a ubiquitous promoter. In the present study, we examined 2 ubiquitous promoters commonly used in mammalian systems: the CMV promoter and the human polypeptide chain elongation factor 1 α (EF-1α) promoter, to determine which promoter is more efficient in hADMPCs. In addition, we also confirmed whether genetically modified hADMPCs maintained their stem cell properties following transduction with this single tet-off lentiviral vector. We examined the expression pattern of cell surface markers, as well as the cells' differentiation potential into adipocytes, chondrocytes, osteocytes, and neuronal cells. Our data demonstrated that hADMPCs transduced with our all-in-one lentiviral vector were effectively selected by blasticidin without affecting their stem cell properties, and transgene expression was strictly regulated by doxycycline (Dox) not only in undifferentiated cells but also in differentiated cells. A single tet-off lentiviral vector system thus provides a powerful tool for applied research on hADMPCs.

## Materials and Methods

### Adipose Tissue Samples

Subcutaneous adipose tissue samples (10–50 g each) were resected during plastic surgery in 5 women (age, 20–60 years) as excess discards. The study protocol was approved by the Review Board for Human Research of Kobe University Graduate School of Medicine, Foundation for Biomedical Research and Innovation, and Kinki University Pharmaceutical Research and Technology Institute (reference number: 10-005). Each subject provided signed informed consent.

### Cell Culture

hADMPCs were isolated as previously reported [1], [11], [25], [26] and maintained in a medium containing 60% DMEM-low glucose, 40% MCDB-201 medium (Sigma Aldrich, St. Louis, MO, USA), 1× insulin-transferrin-selenium (Life technologies, Carlsbad, CA, USA), 1 nM dexamethasone (Sigma Aldrich, St. Louis, MO, USA), 100 mM ascorbic acid 2-phosphate (Wako, Osaka, Japan), 10 ng/mL epidermal growth factor (PeproTech, Rocky Hill, NJ, USA), and 5% fetal bovine serum. The cells were plated to a density of 5×10^3^ cells/cm^2^ on fibronectin-coated dishes, and the medium was replaced every 2 days.

### Plasmid Construction and Lentivirus Production

EGFP was cloned into a pENTR11 vector (Invitrogen) to create an entry vector, pENTR11-EGFP. To generate pTRE-RfA, the tet-responsive element (TRE) of the pTRE-Tight vector (Clontech, Mountain Veiw, CA, USA) and the Reading frame A (RfA), a Gateway cassette containing *att*R recombination sites flanking a *ccd*B gene and a chloramphenicol-resistance gene (Invitrogen) were introduced into *Xba*I-*Xho*I sites of pSico (Addgene plasmid 11578). An improved version of the tet-controlled transactivator (tTA-advanced: pTet-off-advanced Clontech) was linked to the blasticidin resistance (Bsd) gene by the viral T2A peptide to generate tTA-2A-Bsd. Briefly, 2A-Bsd was amplified by PCR using the following primers:

2A-Bsd F: GGGGGATCCGGCGAGGGCAGAGGAAGTCTTCTAACATGCGGTGACGTGGAGGAAAATCCCGGGCCCATGAAGACCTTCAACATCTCTCAG, Bsd R: GCGAGATCTTTAGTTCCTGGTGTACTTG. The resultant product was confirmed by sequencing and ligation with the SmaI site of tTA. EF promoter/CMV promoter and tTA-2A-Bsd was introduced into pTRE-RfA to produce pTRE-RfA-EF-tTA-2A-Bsd or pTRE-RfA-CMV-tTA-2A-Bsd. The entry vector pENTR11-EGFP and pTRE-RfA-EF-tTA-2A-Bsd, pTRE-RfA-CMV-tTA-2A-Bsd, CSII-EF-RfA, or CSII-CMV-RfA (kindly provided by Dr. Miyoshi, RIKEN BioResource Center, Tsukuba, Japan) were incubated with LR clonase II enzyme mix (Invitrogen) to generate pTRE-EGFP-EF-tTA-2A-Bsd, pTRE-EGFP-CMV-tTA-2A-Bsd, CSII-EF-EGFP or CSII-CMV-EGFP. The resultant plasmid was mixed with packaging plasmids (pCAG-HIVg/p and pCMV-VSVG-RSV-Rev, kindly provided by Dr. Miyoshi) and transfected into 293T cells. The supernatant medium, which contained lentiviral vectors, was collected 2 days after transduction and concentrated by centrifugation (6000× *g*, 15 h, 4°C). Viral titers (transduction unit: TU) were determined by serial dilution on 293T cells and the percentage of EGFP positive cells was measured by Guava easyCyte 8HT flow cytometer (Merck-Millipore, Billerica, MA, USA).

### Plasmid Propagation in *E. coli*

DH5α (F-, Φ80dlacZΔM15, Δ(lacZYA-argF)U169, deoR, recA1, endA1, hsdR17(rK-, mK+), phoA, supE44, λ-, thi-1, gyrA96, relA1) were used for general purpose. To propagate plasmids containing the ccdB gene, One Shot® ccdB Survival™ 2 T1 Phage-Resistant (T1R) chemically competent *E. coli* (Invitrogen) were used.

### Western Blot Analysis

Cells were washed with ice-cold phosphate-buffered saline and lysed with M-PER Mammalian Protein Extraction Reagent (Thermo Scientific Pierce, Rockford, IL, USA). Equal amounts of proteins were separated by sodium dodecyl sulfate polyacrylamide gel electrophoresis, transferred to polyvinylidene fluoride membranes (Immobilon-P; Merck-Millipore), and probed with antibody against TetR (from Clontech). Horseradish peroxidase (HRP)-conjugated anti-mouse secondary antibody (Cell Signaling Technology, Danvers, MA, USA) was used as a probe, and immunoreactive bands were visualized with the Immobilon Western Chemiluminescent HRP substrate (Millipore). The band intensity was measured using ImageJ software.

### Flow Cytometry Analysis

hADMPCs were seeded at a density of 2×10^4^ cells per well in 12-well culture plates and were transduced with CSII-EF-EGFP or CSII-CMV-EGFP at a multiplicity of infection (m.o.i.) of 25, 50, 100, 250, 500, and 1000. Four days later, the cells were analyzed with a Guava easyCyte 8HT flow cytometer (Merck-Millipore) using an argon laser at 488 nm. Dead cells were excluded with the LIVE/DEAD fixable far red dead cell stain kit (Invitrogen). For analysis of hADMPCs transduced with pTRE-EGFP-EF-tTA-2A-Bsd or pTRE-EGFP-CMV-tTA-2A-Bsd, hADMPCs were transduced with the lentiviral vector at a m.o.i. of 250 and were cultured with or without 1 µg/mL Dox. Four days later, a part of the cells were analyzed with a Guava easyCyte 8HT flow cytometer. The rest of the cells were cultured with 4 µg/mL blasticidin and 1 µg/mL Dox for 3 weeks. Then, the cells were seeded in 6-well plates and cultured with or without Dox for 4 days. The cells were harvested and re-suspended in staining buffer (PBS containing 1% BSA, 2 mM EDTA, and 0.01% sodium azide) at a density of 1×10^6^ cells/mL and incubated with phycoerythrin (PE)-conjugated antibody against CD13, CD29, CD34, CD44, CD73, CD90, CD105, or CD166 for 20 min. Non-specific staining was assessed using relevant isotype controls. 525/30 nm and 583/26 nm band pass filters were used for the detection of EGFP and PE, respectively. Dead cells were excluded with the LIVE/DEAD fixable far red dead cell stain kit (Invitrogen). FlowJo software (TreeStar Inc., Ashland, OR, USA) was used for quantitation analysis. The threshold for gating was determined as the fluorescence value above which less than 1% of the control cells were considered as positive events.

### Fluorescence Microscopy

Phase contrast and fluorescence images were obtained using Fluorescence Microscope (BZ-9000; Keyence, Osaka, Japan) using BZ Analyzer Software (Keyence).

### Adipogenic, Osteogenic, Chondrogenic, and Neurogenic Differentiation Procedures

For adipogenic differentiation, cells were cultured in differentiation medium (Zen-Bio, Durham, NC, USA). After 3 days, half of the medium was changed to adipocyte medium (Zen-Bio), and this was repeated every 3 days. Three weeks after differentiation, characterization of adipocytes was confirmed by microscopic observation of intracellular lipid droplets by oil red O staining. Osteogenic differentiation was induced by culturing the cells in DMEM containing 10 nM dexamethasone, 50 mg/dL ascorbic acid 2-phosphate, 10 mM β-glycerophosphate (Sigma), and 10% FBS. Differentiation was examined by alizarin red staining. For chondrogenic differentiation, 2×10^5^ hADMPCs were centrifuged at 400× *g* for 10 min. The resulting pellets were cultured in chondrogenic medium (α-MEM supplemented with 10 ng/mL transforming growth factor-β, 10 nM dexamethasone, 100 mM ascorbate, and 1× insulin–transferrin–selenium solution) for 14 days, as described previously [27]. The pellets were fixed with 4% paraformaldehyde in PBS, embedded in OCT, frozen, and sectioned at 8 µm. The sections were incubated with PBSMT (PBS containing 0.1% Triton X-100, 2% skim milk) for 1 h at room temperature, and then incubated with mouse monoclonal antibody against type II collagen (Abcam, Cambridge, MA, USA) and rabbit polyclonal antibody against GFP (Invitrogen) for 1 h. After washing with PBS, cells were incubated with Alexa 546 conjugated anti-mouse IgG and Alexa 488 conjugated anti-rabbit IgG for chondrocytes (Invitrogen) or Alexa 546 conjugated anti-rabbit IgG and Alexa 488 conjugated anti-rat IgG (Invitrogen) for neuronal cells. The cells were counterstained with 4′-6-diamidino-2-phenylindole (DAPI) (Invitrogen) to identify cellular nuclei. For neurogenic differentiation, cells were cultured in Hyclone AdvanceSTEM neural differentiation medium (Thermo Scientific, South Logan, UT, USA) for 2 days. Differentiation was examined by immunofluorescent staining against β3-tubulin. Cells were fixed with 4% paraformaldehyde in PBS for 10 min at 4°C and then washed 3 times in PBS. Blocking was performed with PBSMT for 1 h at room temperature. The differentiated cells were incubated with rabbit monoclonal antibody against β3-tubulin (Cell Signaling Technologies, Danvers, MA, USA) and rat monoclonal antibody against GFP (Nacalai, Kyoto, Japan). After washing with PBS, cells were incubated with Alexa 546 conjugated anti-rabbit IgG and Alexa 488 conjugated anti-rat IgG (Invitrogen). The cells were counterstained with 4′-6-diamidino-2-phenylindole (DAPI) (Invitrogen) to identify cellular nuclei.

## Results

### The Efficiency of the EF-1α Promoter was Higher than that of the CMV Promoter in hADMPCs

**Figure 1:**
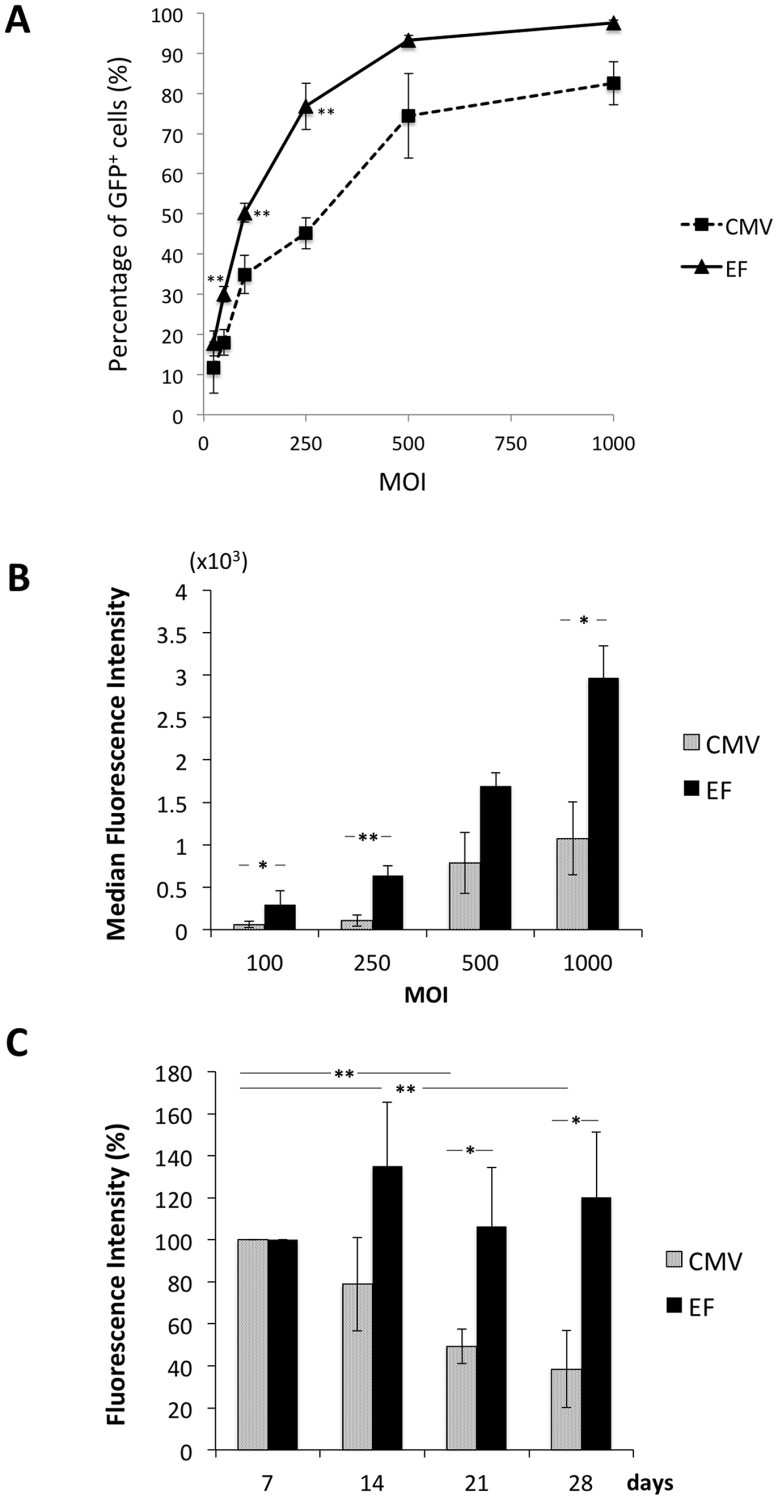
The efficiency of CMV or EF-1α promoter in hADMPCs. Lentiviral vectors encoding EGFP under the control of CMV or EF-1α promoter were transduced with hADMPCs at m.o.i. of 25, 50, 100, 250, 500, and 1000, and the cells were analyzed by flow cytometry. (A) The percentage of EGFP-positive hADMPCs transduced with CSII-CMV-EGFP (CMV) or CSII-EF-EGFP (EF). (B) (C) The median fluorescence intensities of the EGFP-expressing populations. (B) hADMPCs transduced with CSII-CMV-EGFP or CSII-EF-EGFP at m.o.i. of 100, 250, 500, and 1000 were analyzed. (C) hADMPCs transduced with CSII-CMV-EGFP or CSII-EF-EGFP at m.o.i. of 1000 were analyzed over a 28 day period. Error bars represent the standard error of 3 independent analyses. **, P<0.01; *, P<0.05 (Student's t test).

To determine the efficiency of the EF-1α promoter and the CMV promoter, hADMPCs were transduced with CSII-EF-EGFP or CSII-CMV-EGFP at a m.o.i. of 25, 50, 100, 250, 500, and 1000 and analyzed by flow cytometry. As shown in Figure 1A, percentage of GFP-positive cells increased in a dose-dependent manner. Intriguingly, transduction efficiency of CSII-EF-EGFP was significantly higher than that of CSII-CMV-EGFP in hADMPCs (Figure 1A). Moreover, a higher induction level of GFP was observed under the EF-1α promoter than under the CMV promoter, based on the median fluorescent intensity (Figure 1B). Furthermore, GFP fluorescent intensities driven from the CMV promoter were significantly decreased (from 100% on day 7 to 49.3% on day 21 and 38.4% on day 28; Figure 1C), indicating that promoter silencing occurred as previously reported [19]. In contrast, hADMPCs transduced with CSII-EF-EGFP sustained GFP expression levels with no significant reduction throughout the 28-day experimental period (Figure 1C).

### Construction and Characterization of Dual-promoter Lentiviral Vectors in hADMPCs

**Figure 2:**
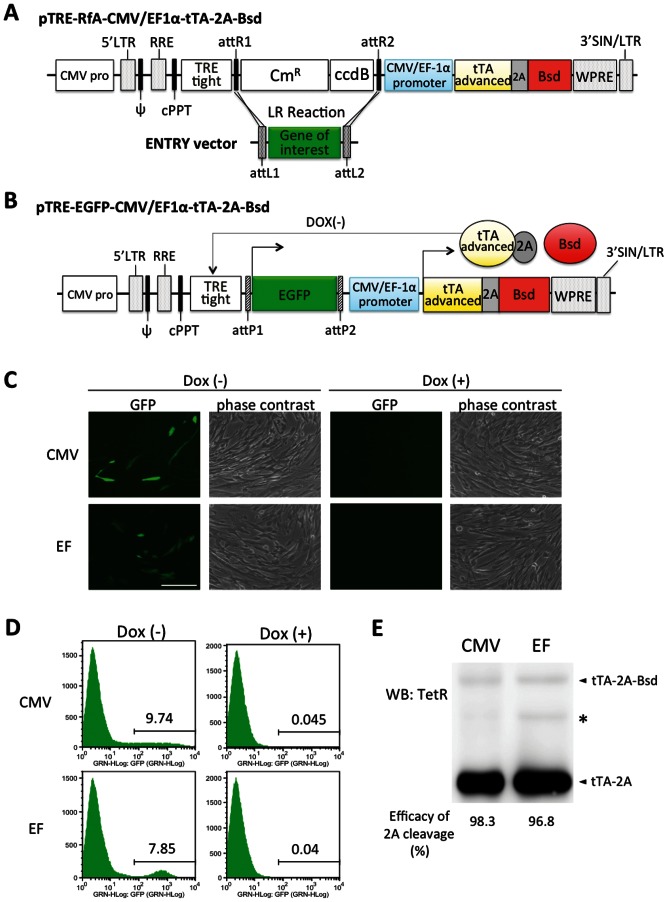
Schematic drawings of the single lentiviral vectors for tet-off system used in this work. (A) Gateway-compatible destination vectors containing *att*R recombination sites flanking a *ccd*B gene and a chloramphenicol-resistance gene, which allows an easy and rapid shuttling of gene of interest flanked by attL sites into the destination vectors using the Gateway LR recombination reaction. They also have an improved version of tetracycline-controlled transactivator (tTA) linked to the blasticidin resistant (Bsd) gene by the Thosea asigna virus 2A (2A) peptide sequence, whose expression is regulated by the CMV or EF-1α promoter. In the present study, we constructed an entry vector encoding EGFP flanked by attL, resulting in a destination clone, pTRE-EGFP-CMV-tTA-2A-Bsd or pTRE-EGFP-EF-tTA-2A-Bsd (B). In the absence of doxycycline (Dox), tTA-2A binds to the TRE-Tight promoter and activates EGFP transcription. For more details, see the Results section. CMV pro, CMV promoter; LTR, long terminal repeats; ψ, packaging signal; RRE, rev response elements; cPPT, central polypurine tract; TRE, tet-responsive element; Cm^R^, chloramphenicol resistance; tTA, tetracycline-controlled transactivator; Bsd, blasticidin resistance; WPRE, woodchuck hepatitis virus posttranscriptional control element; SIN, self-inactivating. (C) (D) hADMPCs were transduced with pTRE-EGFP-CMV-tTA-2A-Bsd or pTRE-EGFP-EF-tTA-2A-Bsd at m.o.i. of 250. Four days after transduction, the cells were divided into 2 populations; with 1 µg/mL of Dox (Dox (+)) and without Dox (Dox (-)). (C) Fluorescent and phase contrast images. Scale bar, 200 µm. (D) Log fluorescence histograms of EGFP by flow cytometry analysis. (E) The whole cell lysates from hADMPCs transduced with pTRE-EGFP-CMV-tTA-2A-Bsd or pTRE-EGFP-EF-tTA-2A-Bsd were subjected to western blotting to monitor the cleavage efficiency of tTA-2A-Bsd proteins. A primary antibody against TetR was used to detect either tTA-2A-Bsd (non-cleaved form) or tTA-2A (cleaved form). Asterisk indicates a nonspecific band.

Next, we constructed dual-promoter lentiviral vectors, which contain TRE-Tight followed by an improved version of tet-controlled transactivator (tTA advanced) induced under the CMV or EF-1α promoter (Figure 2A). In this “single tet-off lentiviral vector platform”, the regulator and response elements are combined in a single lentiviral genome, along with a Gateway cassette containing *att*R recombination sites flanking a *ccd*B gene and a chloramphenicol-resistance gene, which allows an easy and rapid shuttling of the gene of interest into the vectors using the Gateway LR recombination reaction (Figure 2A). Using this system, we constructed pTRE-EGFP-CMV-tTA-2A-Bsd or pTRE-EGFP-EF-tTA-2A-Bsd (Figure 2B). Both the CMV and the EF-1α promoters drive the mRNA expression of tTA advanced linked to the Bsd gene by the Thosea asigna virus 2A (T2A) peptide sequence. This single transcript is then translated and cleaved into 2 proteins; tTA advanced carrying 2A tag at the C-terminus (tTA-2A) and Bsd. tTA-2A binds to the TRE-tight in the absence of Dox, a tet derivative, and activates transcription of EGFP to a very high level. In the presence of Dox, tTA-2A is unable to bind the TRE-Tight in a tet-responsive promoter, and the system is inactive.

To investigate the usefulness of these lentiviral vectors, hADMPCs were transduced with pTRE-EGFP-CMV-tTA-2A-Bsd or pTRE-EGFP-EF-tTA-2A-Bsd at a m.o.i. of 250. As shown in Figure 2C, expression of EGFP was observed in the absence of Dox, whereas addition of Dox (1 µg/mL) was enough to suppress the expression. Flow cytometry analysis revealed that the transduction efficiency was relatively low (EGFP-positive cells were 7.5∼10%) compared with that of CSII-CMV-EGFP or CSII-EF-EGFP (EGFP-positive cells were 45% or 77% at a m.o.i. of 250, respectively; Figure 1A), and the tet-off system completely abolished gene expression in the presence of Dox (Figure 2D). Flow cytometry analysis also revealed that fluorescent intensity was relatively uniform in hADMPCs transduced with pTRE-EGFP-EF-tTA-2A-Bsd, but a wide range of fluorescent intensities was observed in hADMPCs infected with pTRE-EGFP-CMV-tTA-2A-Bsd. These data suggest that tTA-2A functions properly in this system. Moreover, western blot analysis against tTA showed the efficient cleavage (>95%) of tTA-2A-Bsd proteins into tTA-2A and Bsd (Figure 2E).

**Figure 3:**
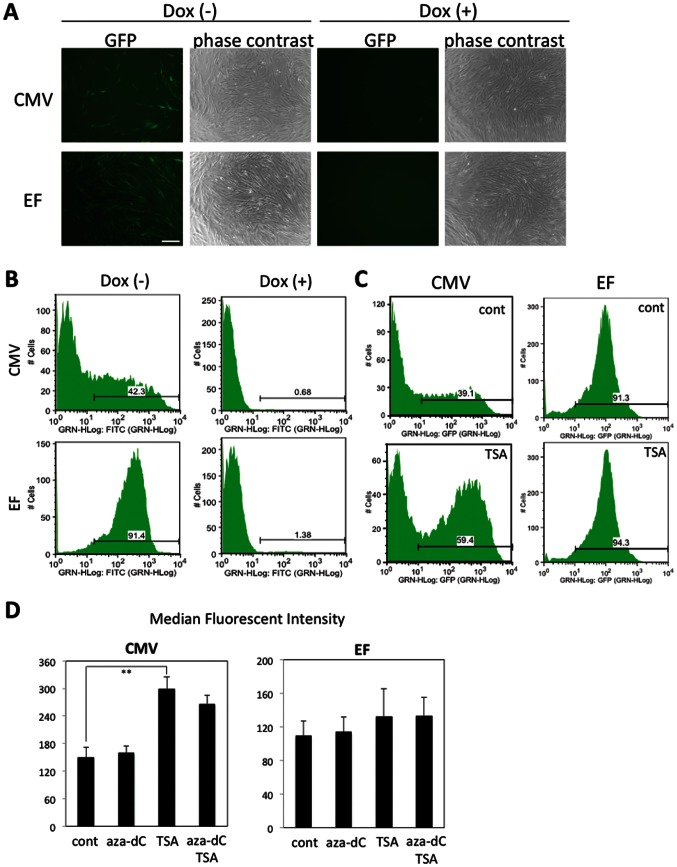
Blasticidin selection of hADMPCs transduced with single tet-off lentiviral vector platform. hADMPCs were transduced with pTRE-EGFP-CMV-tTA-2A-Bsd (CMV) or pTRE-EGFP-EF-tTA-2A-Bsd (EF) at m.o.i. of 250. The cells were treated with 4 µg/mL blasticidin and 1 µg/mL Dox for 2 weeks. Then, the cells were cultured in the absence (Dox (-)) or presence (Dox (+)) of 1 µg/mL Dox for 4 days, and analyzed under a microscope (A) and flow cytometer (B). The cells were treated with 100 nM TSA (TSA), 5 µM 5-aza-dC (aza-dC), or both for 48 h before analyzed by flow cytometer. (C) A representative fluorescence histogram of EGFP. (D) The median fluorescence intensities of the EGFP-expressing populations. Error bars represent the standard error of 3 independent analyses. **, P<0.01 (Student's t test). Scale bar, 200 µm.

To further determine that Bsd cleaved from tTA-2A-Bsd was effective in this system, 4 µg/mL blasticidin was administered to hADMPCs. Within 1 week after the selection, control hADMPCs were completely killed (data not shown), whereas hADMPCs that were successfully transduced with either pTRE-EGFP-CMV-tTA-2A-Bsd or pTRE-EGFP-EF-tTA-2A-Bsd could survive and proliferate, demonstrating that Bsd from tTA-2A-Bsd is sufficient to confer blasticidin resistance to the cells. The surviving cells were kept in culture medium with blasticidin and then divided into 2 populations, either with Dox (1 µg/mL) or without Dox. As shown in Figure 3A and 3B, almost all (>90%) the cells transduced with pTRE-EGFP-EF-tTA-2A-Bsd strongly expressed EGFP in the absence of Dox. In hADMPCs transduced with pTRE-EGFP-CMV-tTA-2A-Bsd, however, >50% of the cells were EGFP negative regardless of their blasticidin resistance. Moreover, fluorescent intensities were quite variable; some cells expressed very high levels of EGFP, while others expressed very low levels (Figure 3A and 3B). This might be due to “promoter suppression,” transcript repression of an upstream transcriptional unit by a downstream unit when 2 transcriptional units lie adjacent in head-to-tail tandem on a chromosome [28], [29]. Studies have revealed that the suppression by adjacent units is epigenetic and involves modification of the chromatin structure, including DNA methylation at CpG sites within the promoter, histone deacetylation, histone methylation at specific residues (e.g., H3K9, H3K27), and densely packed nucleosomes that create a closed chromatin structure. In order to determine if inhibiting histone deacetylases or DNA methylation would re-induce EGFP expression, pTRE-EGFP-CMV-tTA-2A-Bsd cells were treated with histone deacetylase inhibitor trichostatin A (TSA) and/or DNA methylation inhibitor 5-aza-2′-deoxycytidine (5-aza-dC). TSA treatment significantly increased the number of EGFP-positive cells and strengthened the fluorescent intensities of EGFP, whereas 5-aza-dC had no effect, suggesting that EGFP expression was repressed by histone deacetylation when stably transduced with pTRE-EGFP-CMV-tTA-2A-Bsd (Figure 3C and 3D). These inhibitors had no effect on hADMPCs transduced with pTRE-EGFP-EF-tTA-2A-Bsd. These data suggest that the dual-promoter lentiviral vector using the EF promoter is more resistant to gene silencing than that using the CMV promoter.

### Blasticidin-selected hADMPCs Maintain the Properties of Their Parental hADMPCs

**Figure 4:**
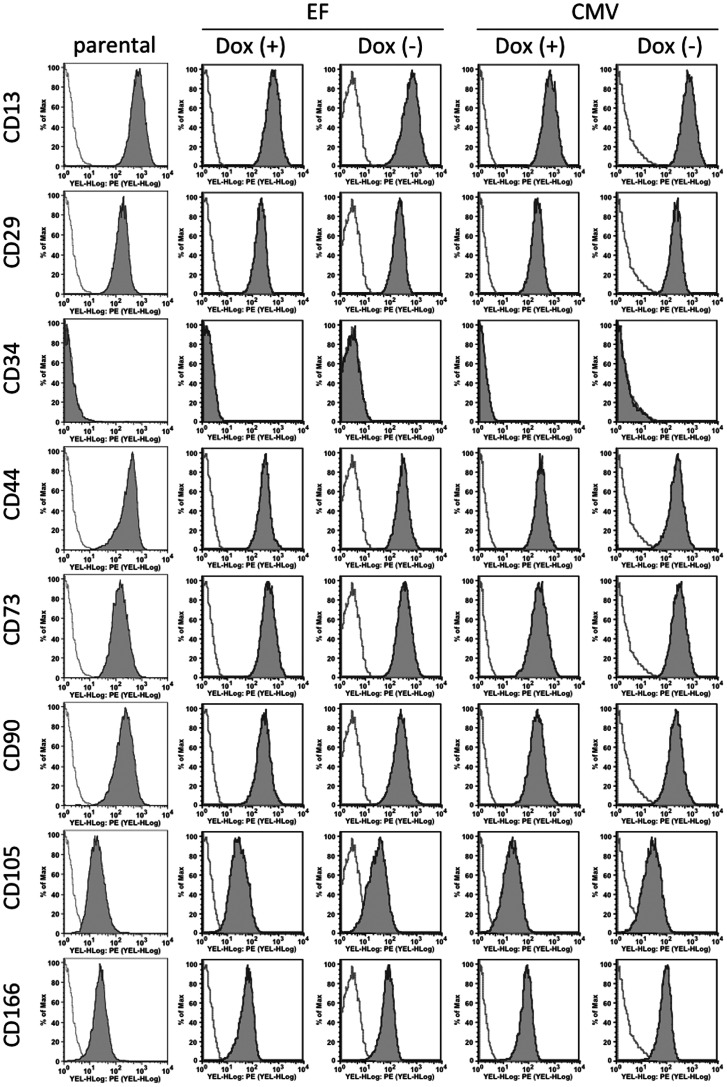
Expression pattern of surface cell markers on Dox-responsive hADMPCs. Dox-responsive hADMPCs after selection by blasticidin were cultured in the absence (Dox(-)) or presence (Dox (+)) of 1 µg/mL Dox for 4 days. Expression of the different surface markers were analyzed by flow cytometry and compared to the expression by a parental hADMPCs. They were stained with PE-coupled antibodies against CD13, CD29, CD34, CD44, CD73, CD90, CD105, and CD166. Histogram of a PE-coupled mouse IgG1 κ isotype control is shown in gray. CMV; hADMPCs transduced with pTRE-EGFP-CMV-tTA-2A-Bsd, EF; hADMPCs transduced with pTRE-EGFP-EF-tTA-2A-Bsd.

**Figure 5:**
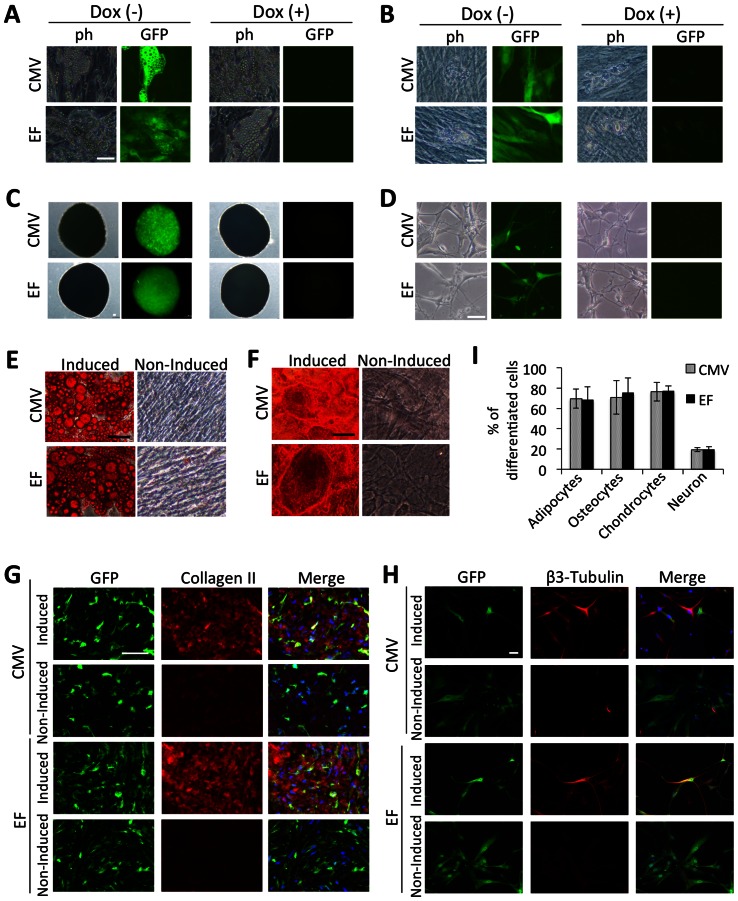
Differentiation potential of Dox-responsive hADMPCs. Dox-responsive hADMPCs were differentiated into adipocytes (A, E), osteocytes (B, F), chondrocytes (C, G), and neuronal cells (D, H). (A–D) Phase contrast (ph) and fluorescent (GFP) images. Dox-responsive hADMPCs were differentiated in the absence of Dox (Dox(−)) or in the presence of 1 µg/mL Dox (Dox(+)) as described in the material and methods section. (E–I) Confirmation of differentiated cells by oil red O staining for adipocytes (E), alizarin red staining for osteocytes (F), immunohistochemical staining against collagen II for chondrocytes (G), and immunohistochemical staining against β3-tubulin for neuronal cells (H). The percentages of differentiated cells to each cell type were calculated by the computerized image analysis (I). Cells that were not induced to differentiate (non-induced) were used as a negative control. CMV; hADMPCs transduced with pTRE-EGFP-CMV-tTA-2A-Bsd, EF; hADMPCs transduced with pTRE-EGFP-EF-tTA-2A-Bsd. Scale bar, 50 µm.

hADMPCs are an attractive material for cell therapy because of their ability to secrete various cytokines and growth factors. These cells also have the ability to differentiate into various types of cells, including adipocytes, chondrocytes, osteocytes, hepatocytes, cardiomyoblasts, and neuronal cells. Gene manipulation of hADMPCs may thus generate great possibilities for cell therapy and tissue engineering. From this point of view, the development of an efficient and stable Dox-responsive gene transfer system to achieve high levels of transgene expression in hADMPCs, without affecting the phenotype, is of special interest for the field. We therefore studied the cell properties of hADMPCs transduced with the single tet-off lentiviral vector after blasticidin selection. Flow cytometry analysis revealed no changes in the expression of the main surface markers (positive for CD13, CD29, CD44, CD73, CD90, CD105, and CD166, and negative for CD34) either in the absence or presence of Dox (Figure 4). To further confirm the properties of hADMPCs, the cells were differentiated into adipocytes, osteocytes, chondrocytes, and neuronal cells. As shown in Figure 5, blasticidin-selected hADMPCs maintained their ability to differentiate into adipocytes, osteocytes, chondrocytes, and neuronal cells. Moreover, EGFP was stably expressed in the differentiated cells only in the absence of Dox (Figure 5).

## Discussion

In recent years, there is growing interest in the use of MSCs for cell therapy and tissue engineering because of their differentiation potential and ability to secrete growth factors [7]–[11]. Furthermore, because of their hypo-immunogenicity and immune modulatory effects, MSCs are good candidates for gene delivery vehicles for therapeutic purposes [12], [14]. In addition to primary MSCs, genetically modified MSCs have been applied to bone regeneration, muscle repair, diabetes, Parkinson's disease, and myocardial infarction recovery [14], [30]–[35]. Duan et al. reported that the angiogenic effect of MSCs could be enhanced by adenovirus-mediated HGF overexpression in the treatment of cardiac ischemia injury [14]. Karnieli et al. and Li et al. both reported the reversal of hyperglycemia in streptozotocin-induced diabetic mice after transplantation of insulin-producing cells originating from genetically modified Pdx-1 expressing MSCs [32], [33].

While significant progress has been made in the use of genetically modified MSCs for basic and applied research, the current methods for gene manipulation are still insufficient for some applications. Adenoviral vectors are commonly used for transient expression because they remain epichromosomal in the host cells, and their ability to transiently infect target cells minimizes the risk of insertional mutagenesis [36]. However, relatively brief transgene expression may limit the utility of this approach to tissue repair applications. On the other hand, lentiviral vectors, which are promising vectors for gene delivery in primary human cells, integrate into the host cell genome, which may be an appropriate strategy for tissue repair applications requiring sustained, long-term expression of therapeutic proteins. In this study, we generated novel lentiviral vectors with a tet-off system, and demonstrated that our lentiviral vector systems were significantly effective and strictly regulated in hADMPCs, without affecting their stem cell properties.

Gene silencing is of considerable importance where stable, long-term expression is required. Researchers have reported that transgene silencing occurred when the CMV promoter was used in some cell types, especially in embryonic stem cells [15]–[17]. Since Kawabata et al. also demonstrated that virus-derived promoters inefficiently functioned in embryonic stem cells in gene transfer experiments [37], down-regulation and unsuitability of promoters in stem cells should be considered. Therefore, transduction efficacy and durability of transgene expression in hADMPCs is also an important issue to be determined. Qin et al. reported that the human EF-1α promoter and the TRE promoter are more efficient than the CMV promoter to drive lentiviral mediated transgene expression in rat bone marrow-derived MSCs [18]. McGinley et al. also showed that EF-1α and human phosphoglycerate kinase-1 (PGK) promoters have a clear advantage over the CMV promoter in transducing rat bone marrow-derived MSC transduction with lentivirus [19]. Consistent with their findings, our data also demonstrated that the EF-1α promoter was more efficient than the CMV promoter to drive EGFP expression in hADMPCs (Figure 1A, B). Moreover, a significant decrease in fluorescent intensity was observed by 28 days after transduction with lentiviral vector CSII-CMV-EGFP (Figure 1C), suggesting that the CMV promoter might be silenced in hADMPCs. We also demonstrated the intriguing finding that most (>90%) of the hADMPCs transduced with pTRE-EGFP-EF-tTA-2A-Bsd strongly expressed EGFP in the absence of Dox, whereas >50% of the cells transduced with pTRE-EGFP-CMV-tTA-2A-Bsd were EGFP negative, regardless of their blasticidin resistance (Figure 3A, B). Our data demonstrated that the inhibitor of histone deacetylation trichostatin A (TSA) re-induced the expression of EGFP (Figure 3C, D), suggesting that “promoter suppression” might occur by histone deacetylation, not by DNA methylation of CpG sites within the TRE tight promoter. “Promoter suppression” is a transcript repression of a 5′ transcriptional unit by a 3′ unit when 2 transcriptional units lie adjacent in head-to-tail tandem on a chromosome [28], [29]. In this study, it is possible that the downstream unit of CMV-tTA-2A-Bsd repressed the upstream unit of TRE-EGFP because (1) resistance to blasticidin implies the transcriptional unit of CMV-tTA-2A-Bsd is active, and (2) reactivation of EGFP expression by TSA implies the transcriptional unit of TRE-EGFP is epigenetically silenced. In order to eliminate the promoter suppression or transcriptional interference between 2 transcriptional units, some researchers have been trying to separate the 2 units by polyadenylation, terminator, and insulator sequences [28], [38]. However, these sequences extend the lentiviral vector size, which may affect the lentiviral titers produced from the vector. From this point of view, our finding that the transcriptional unit driven from the TRE tight promoter is resistant to gene silencing when arranged in tandem with the EF-tTA-2A-Bsd transcriptional unit (Figure 3) is of interest in the fields of both basic and clinical research, although the underlying mechanism remains elusive.

In general, large numbers of cells displaying the appropriate phenotypes are required for tissue engineering. Moreover, fully differentiated cells do not proliferate [39]. Therefore, in order to obtain enough cells to perform a transplant from genetically modified MSCs, it is important to develop a system in which the gene of interest is tightly regulated and inducible, and in which stably expressing transgenic cell lines can be obtained without affecting their stem cell properties. Using the system, MSCs transduced with lentiviral vectors can be selected and increased in numbers from a limited number of MSCs, before the target genes are induced. After obtaining an adequate number of gene-manipulated MSCs, the target genes could be induced in order to start differentiation. According to our data, hADMPCs transduced with pTRE-EGFP-EF-tTA-2A-Bsd were successfully selected by blasticidin, could proliferate, maintain their stem cell properties, and regulate EGFP expression tightly by Dox (Figure 4, 5), demonstrating that this all-in-one lentiviral vector is a promising gene delivery system for generating the material for artificial organs.

A major advantage of using the 2A cleavage factor in the construction of multi-cistronic vectors is its small size compared to internal promoter entry site (IRES) sequences. Because the titer of the lentivirus decreases with increasing size of the lentiviral vector, it is important to minimize the length of the sequences. In addition, linkage of 2 genes by 2A peptide resulted in efficient co-expression of the genes, whereas a gene placed downstream of an IRES is expressed at 2- to 3-fold lower levels than a gene placed upstream [40], [41]. In this study, tTA-2A-Bsd cassette driven from CMV or EF-1α promoter showed ∼90% cleavage (Figure 3). However, the point that should be considered is the effect of residual 2A peptide on the protein. As the processing occurred at the end of the 2A peptide, the 2A tag remains attached at the tTA C-terminus. Our data demonstrated that the presence of this extra 2A peptide did not seem to interfere with the activity of tTA since Dox strictly regulated the expression of EGFP under the control of TRE-tight promoter (Figure 2D, 3A, 3B and 5). Moreover, when Bsd is cleaved, an additional proline is attached at the N-terminus. We demonstrated that this did not affect a function of Bsd because hADMPCs transduced with either pTRE-EGFP-CMV-tTA-2A-Bsd or pTRE-EGFP-EF-tTA-2A-Bsd could survive and proliferate in medium containing blasticidin at a concentration at which all of the parental hADMPCs died.

Another advantage of our lentiviral system is the availability of a restriction enzyme treatment/ligation independent cloning system, called the Gateway system (Invitrogen). In general, the construction of lentiviral vectors using a conventional restriction enzyme/ligation cloning method has poor efficiency due to the large sizes and the lack of proper cloning sites. In our hands, cloning efficiency into our new lentiviral vectors pTRE-RfA-CMV-tTA-2A-Bsd or pTRE-RfA-EF-tTA-2A-Bsd using LR recombination reaches nearly 100%, saving time and effort in construction of the vectors. In addition, there are several resources available that take advantage of the Gateway vector. For example, CCSB Human ORFeome Collection (Dana-Farber Cancer Institute, Center for Cancer Systems Biology) represents almost 12,000 fully-sequenced cloned human ORFs which can be readily transferred to Gateway compatible destination vectors for various functional proteomics studies [42]. Block-iT pol II miR RNAi system from Invitrogen, which is designed to express artificial miRNAs, also enables compatibility with Gateway destination vectors for gene knockdown experiments [43].

In conclusion, our new single tet-off lentiviral vector system provides powerful tools not only for applied research on hADMPCs and other stem cells, but also basic research on a variety of cell lines and primary cells.
